# Assessing Cerebrovascular Reactivity in Carotid Steno-Occlusive Disease Using MRI BOLD and ASL Techniques

**DOI:** 10.1155/2012/268483

**Published:** 2012-06-20

**Authors:** Renata F. Leoni, Kelley C. Mazzetto-Betti, Afonso C. Silva, Antonio C. dos Santos, Draulio B. de Araujo, João P. Leite, Octavio M. Pontes-Neto

**Affiliations:** ^1^Department of Neuroscience and Behavioral Sciences, FMRP, University of Sao Paulo, Ribeirao Preto, SP 14049-900, Brazil; ^2^Cerebral Microcirculation Unit, Laboratory of Functional and Molecular Imaging, National Institute of Neurological Disorders and Stroke, NIH, Bethesda, MD 20892, USA; ^3^Onofre Lopes University Hospital, Federal University of Rio Grande do Norte, Natal, RN 59012-300, Brazil; ^4^Brain Institute, Federal University of Rio Grande do Norte, Natal, RN 59020-130, Brazil

## Abstract

Impaired cerebrovascular reactivity (CVR), a predictive factor of imminent stroke, has been shown to be associated with carotid steno-occlusive disease. Magnetic resonance imaging (MRI) techniques, such as blood oxygenation level-dependent (BOLD) and arterial spin labeling (ASL), have emerged as promising noninvasive tools to evaluate altered CVR with whole-brain coverage, when combined with a vasoactive stimulus, such as respiratory task or injection of acetazolamide. Under normal cerebrovascular conditions, CVR has been shown to be globally and homogenously distributed between hemispheres, but with differences among cerebral regions. Such differences can be explained by anatomical specificities and different biochemical mechanisms responsible for vascular regulation. In patients with carotid steno-occlusive disease, studies have shown that MRI techniques can detect impaired CVR in brain tissue supplied by the affected artery. Moreover, resulting CVR estimations have been well correlated to those obtained with more established techniques, indicating that BOLD and ASL are robust and reliable methods to assess CVR in patients with cerebrovascular diseases. Therefore, the present paper aims to review recent studies which use BOLD and ASL to evaluate CVR, in healthy individuals and in patients with carotid steno-occlusive disease, providing a source of information regarding the obtained results and the methodological difficulties.

## 1. Introduction

Cerebrovascular reactivity (CVR) is a unique physiologic characteristic of the brain related to the intrinsic ability of encephalic arteries to alter their caliber in response to a vasoactive stimulus [1]. Impaired CVR has been shown to be associated with different pathological conditions, such as hypertension [2–4], arterial stenosis [5–7], and proximal arterial occlusion [8–11]. Severe arterial stenosis or occlusion of proximal vessels of the neck may reduce perfusion pressure in ipsilateral brain regions when collateral flow is insufficient, resulting in autoregulatory vasodilation to maintain regional cerebral blood flow (CBF) within normal limits and then exhausted cerebrovascular reserve capacity, which might contribute to a higher risk of ischemic stroke [12, 13]. Therefore, investigation of altered CVR may be predictive of imminent stroke in such patients [9, 14–16]. 

CVR has been generally assessed by positron emission tomography (PET) [17, 18], single-photon emission computed tomography (SPECT) [19, 20], and transcranial Doppler (TCD) ultrasonography [21–23]. These methods investigate the residual capacity of cerebral arteries to dilate in response to an increase of carbon dioxide (CO_2_), via CO_2_ inhalation, breath-holding test (BHT), or acetazolamide (ACZ) administration. 

The ability to quantify regional CBF, cerebral blood volume (CBV), and oxygen extraction fraction (OEF) makes PET imaging the gold standard for cerebral perfusion evaluation [24]. In healthy subjects, regional differences in CVR have been reported, indicating regional differences in cerebral vascular tone [25, 26]. Changes in CBF and vasodilatory capacity have been also assessed in patients with cerebral artery occlusion, and results indicated critical hemodynamic status in these patients [10, 27]. However, although PET has been shown to be useful for CVR assessment, the high cost, technical complexity, limited spatial resolution, and exposure to radiation preclude its routine clinical use. 

SPECT, another technique that allows CBF quantification, has also been used to investigate CVR. Studies have shown that reduced regional CVR resulting from arterial occlusion is associated with higher risk of ischemic stroke [14, 15]. However, this technique requires repeated measurements, before and after ACZ administration, which increases exposure to radiation and introduces errors due to head motion artifacts and heterogeneous washout of tracer [28].

An alternative to either PET or SPECT in the evaluation of CVR is TCD ultrasonography, a simple and noninvasive technique that allows rapid measurements of flow velocities in large cerebral arteries. With a vasodilatory stimulus, it has also provided CVR assessment in healthy subjects and in patients with carotid artery steno-occlusive disease [1, 9, 16, 22, 29–31]. Studies using TCD have also suggested that impaired CVR is predictive of cerebral ischemic events in such patients [9, 32]. However, lack or poor insonation of the transcranial window may prevent measurements in some subjects [9]. Moreover, despite its high temporal resolution, low cost and reproducibility, TCD's low spatial resolution and, consequently, lack of regional specificity, limits brain mapping. 

In the past 10–15 years, magnetic resonance imaging (MRI) techniques have emerged as promising tools to evaluate cerebral hemodynamics with whole-brain coverage. Blood oxygenation level-dependent (BOLD) images can be acquired with the same vasoactive stimulus cited above, and the percentage of signal change can be used to assess CVR [33, 34]. Furthermore, the arterial spin labeling (ASL) technique allows CBF measurements at rest and under a vasoactive stimulus, providing quantitative measurements of CVR [4, 35, 36]. In the present paper, a review of several studies which use BOLD and ASL to evaluate CVR, in healthy individuals and in patients with arterial steno-occlusive disease, is presented to provide a useful source of critical information for future improvements on this field. 

## 2. Vasoactive Challenges to Map CVR

It has been shown that CO_2_ has profound effects on cerebrovascular tone, resulting in CBF changes [37–39]. However, arterioles in an injured brain may be less responsive to CO_2_ alterations, reducing CBF changes. In patients with occlusive cerebrovascular disease, CVR is generally impaired. Therefore, hypo- and hyper-capnia can be used for CVR mapping in such patients in order to detect brain areas with altered vasodilatory capacity [5, 9, 16, 26, 31, 35, 40]. However, regional differences should be considered when evaluating CVR in patients, making important the investigation of CVR distribution in the brain of healthy individuals.

Hypercapnia can be achieved by BHT or CO_2_ inhalation, which increase the arterial partial pressure of CO_2_ (PaCO_2_), without changing the cerebral metabolic rate of oxygen (CMRO_2_). The feasibility and efficacy to investigate CVR with different imaging techniques in combination with hypercapnia have been shown in studies of healthy subjects [7, 22, 41] and also of patients with carotid stenosis or occlusion [9, 12, 16]. Both challenges, BHT and CO_2_ inhalation, have provided similar vascular responses [42].

BHT was initially proposed by Ratnatunga and Adiseshiah in 1990 to test cerebral perfusion reserve with TCD [22]. Since then, several studies have used different protocols with BHT to study CVR in healthy subjects and patients. Breath-holding durations from 14 to 30 seconds, after inspiration or expiration, have been reported [7, 8, 16, 43–47]. The main advantage of BHT is that it can be easily performed during a routine MR examination, with no need of exogenous CO_2_ source or ACZ injection. However, despite the good practicability, potential shortcomings should be noted. First, the subjects have to be able to understand and perform the task correctly, making it difficult to be used with patients with cognitive deficits or any other problem that preclude BHT execution. Second, BHT leads to different rise rates of PaCO_2_ in different people, increasing intersubject variability [48]. Moreover, a voluntary act of breath-holding can induce local CBF and oxygenation changes due to neuronal activation. Recently, the use of cue and feedback mechanisms to control initial inspiration and prolonged breath-holding has been proposed in order to keep the desired PaCO_2_ level and reduce intersubject variability [49]. 

On the other hand, CO_2_ inhalation is a passive task, less dependent on subjects' cooperation. Although it needs an additional apparatus to deliver the gas, it is possible to control the hypercapnia level by the administration of CO_2_/air mixture. Fixed CO_2_ concentrations from 3 to 10% have been generally used [9, 40–42, 50, 51]. Although no adverse effects were observed in such studies, depending on the duration of gas inhalation, CO_2_ concentrations greater than 7% were reported to be exhausting [50]. Moreover, CO_2_ inhalation may be not tolerable to elderly subjects and not safe to patients with obstructive pulmonary diseases [5]. The CO_2_ inhalation method makes it convenient to deliver the gas, but individual results vary with differences in metabolism amongst subjects. To minimize variability, a method that uses computer-controlled feedback to rapidly set end-tidal oxygen (O_2_) and CO_2_ concentrations on a breath-by-breath basis to desired target levels was shown to provide a flexible and physiologically well-controlled way to evaluate CVR [52].

Hyperventilation is also a robust and reproducible method to assess CVR. Contrary to BHT and CO_2_ inhalation, it is associated with reduction in CBF. The physiological effect of hyperventilation is to reduce PaCO_2_, increasing pH, which increases cerebrovascular resistance, resulting in marked vasoconstriction and, consequently, reduction in blood flow [34, 37, 53]. A recent respiratory task, named “cued deep breathing” (CDB), has been developed to cause transient mild hypocapnia, and consequently vasoconstriction. Resulting high-quality CVR maps comparable to those acquired using BHT indicate CDB as an alternative method in clinical applications [54].

Another vasodilatory substance is the ACZ, a selective inhibitor of carbonic anhydrase that decreases the conversion rate of CO_2_ to bicarbonate. Injection of ACZ causes CO_2_ retention in brain tissue, changing extracellular pH, and thus rapidly and markedly increasing CBF, leaving CMRO_2_ and arterial blood pressure unchanged [20]. Although invasive, ACZ administration has been used to assess CVR in healthy subjects [1, 7] and in patients with cerebrovascular disorders [10, 14, 15, 19]. In a TCD study, vasodilatory response to ACZ was comparable to the response to BHT and CO_2_ inhalation [21]. However, another study showed that CO_2_ inhalation provides a more reproducible increase in blood flow than ACZ [55]. 

**Figure 1:**
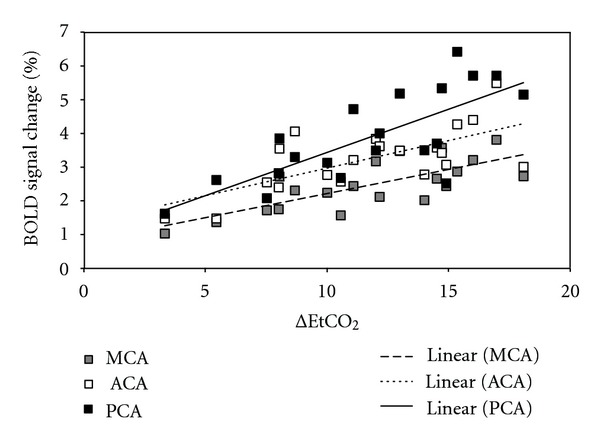
Linear correlation between BOLD signal change and ΔEtCO_2_ in brain regions supplied by the three main cerebral arteries (anterior: ACA, middle: MCA, and posterior: PCA), for a group of healthy subjects who underwent CO_2_ inhalation (*r* ^2^ = 0.73 ± 0.04). Note that the highest increases in BOLD signal were observed in region areas supplies by the posterior cerebral artery (PCA: black squares), and that the lowest BOLD signal amplitudes were observed in areas supplied by the middle cerebral artery (MCA: gray squares).

Some studies have investigated the relationship between blood flow and CO_2_ changes. A linear correlation between BOLD signal changes and the partial pressure of end-tidal CO_2_ (PetCO_2_) was shown in gray and white matter using a breathing device for controlling CO_2_ levels [41]. In an ongoing study of our group, the same linear correlation was observed in brain regions supplied by three main cerebral arteries (Figure 1). However, a method using hyperventilation and rebreathing was employed to obtain a wider range of CO_2_ changes, from hypocapnic to hypercapnic values [29]. Results showed a sigmoidal relationship between CBF and CO_2_, indicating that CBF may reach a plateau for high levels of PaCO_2_, as previously reported in human and nonhuman primates [56, 57]. The linear part of the sigmoidal curve is between PCO_2_ values of 25–65 mmHg [41, 56]. Within this range, CBF normally changes 2–6% per mmHg change in PCO_2_ [25]. 

## 3. CVR Mapping with BOLD

BOLD signal reflects differences on the magnetic susceptibility of intravascular hemoglobin, depending on whether it is bound to oxygen or not. Oxyhemoglobin is diamagnetic, while deoxyhemoglobin is paramagnetic. An increase in regional CBF surpassing an increase in oxygen consumption results in a reduction of deoxyhemoglobin concentration, increasing local signal intensity in T2*-weighted images [58]. Although BOLD images have been mainly used in studies of brain function, they have a great potential to investigate cerebral perfusion and estimate CVR noninvasively, measuring changes in signal intensity after different challenges, such as injection of ACZ [59–61], BHT [5, 42, 45, 51], hyperventilation [34], and CO_2_ inhalation [40–42, 50, 51, 62, 63].

**Figure 2:**
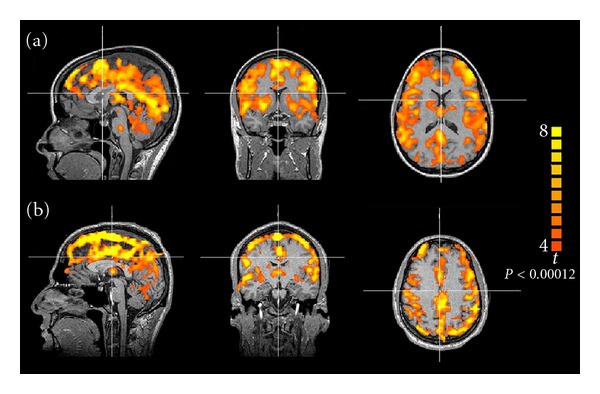
Representative BOLD signal maps showing global response to (a) breath-holding test and (b) CO_2_ inhalation in healthy subjects.

Under normal cerebrovascular conditions, CBF in response to vasodilatory stimulus increases globally and homogeneously between hemispheres, but with differences among cerebral regions. Therefore, global increase in BOLD signal has been observed in response to BHT (Figure 2(a)) or CO_2_ inhalation (Figure 2(b)). Studies using BHT and CO_2_ inhalation have shown significant increase in signal intensity in gray matter, but not in white matter [33, 51, 64, 65]. In one of these studies, inhalation of 8% CO_2_ increased PetCO_2_ from 30 mmHg to 46 mmHg, resulting in an increase of BOLD signal of 4 ± 3% in gray matter and nonsignificant change in white matter [51]. Absence of vasodilation in white matter or insufficient sensitivity of the technique was used to explain that result. However, significant BOLD signal variations in white matter were observed in other studies; although the increase was slower, and the amplitude was lower compared to gray matter [41, 50]. The same results were previously found in a study using hyperventilation; however, there was a decreasing response due to hypocapnia [34]. In a study using 7% CO_2_ inhalation, BOLD signal changes were 5.9 ± 1.2% and 1.9 ± 0.5%, for gray and white matters, respectively [41]. There is evidence that changes in CBF are mainly due to changes in transit time rather than changes in the number and volume of perfused capillaries [66]. Since the transit time of the hemoglobin is higher in white matter [66], it may explain the differences in BOLD signal changes between gray and white matters. 

**Figure 3:**
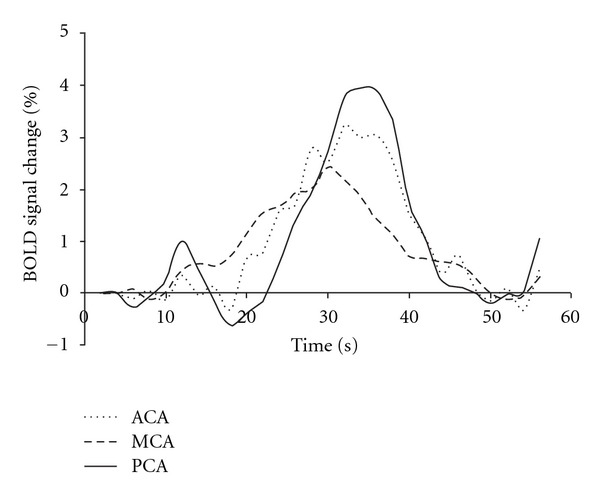
BOLD responses to CO_2_ inhalation in brain regions supplied by the three main cerebral arteries (anterior: ACA, middle: MCA, and posterior: PCA), for healthy subjects.

Regional differences in BOLD responses to a vasoactive stimulus were also observed within gray matter in healthy children and adults [47]. Higher amplitudes were observed in cortical (frontal, occipital, and parieto-occipital cortices) compared to subcortical regions [34, 48]. Within cortical regions, areas supplied by the posterior cerebral artery (PCA), such as the inferior temporal and the occipital gyri, showed the highest BOLD signal amplitudes after BHT or CO_2_ inhalation [45, 50, 67]. On the other hand, areas supplied by the middle cerebral artery (MCA), such as the middle and inferior frontal gyri, inferior parietal lobes and superior and middle temporal gyri, showed the lowest BOLD signal amplitudes [45]. Different BOLD signal onsets were also observed in different brain regions, indicating an apparent temporal evolution of the response [43, 45, 54].  Figure 3 shows BOLD signal responses to CO_2_ inhalation in three different brain regions for a group of healthy subjects. Anatomical specificities, such as capillary density, resting CBV, and different biochemical mechanisms responsible for vascular regulation may explain these results [68, 69]. 

**Figure 4:**
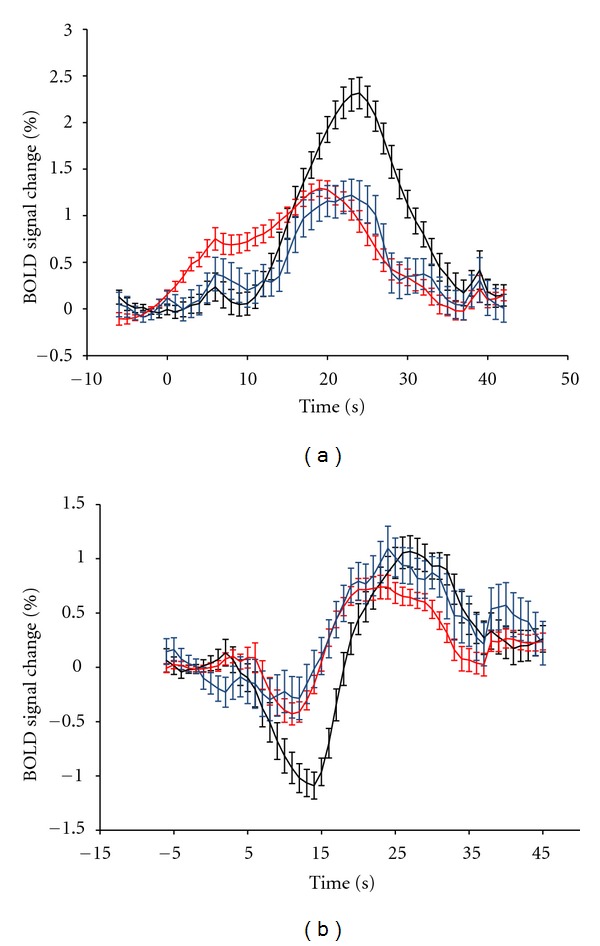
Average BOLD response to breath-holding of 15 seconds after (a) expiration and (b) inspiration on brain regions supplied by the three main cerebral arteries (anterior: blue, middle: red, and posterior: black), for a group of healthy subjects. Reproduced from Leoni et al. [45].

Studies using BHT showed that the shape and the amplitude of BOLD responses are not dependent only on brain region, but also on breath-holding duration and technique. Longer breath-holding results in greater BOLD signal amplitude, since greater CO_2_ levels are presented in blood [43]. Moreover, BOLD responses are dependent on breath-holding technique (Figure 4). Breath-holding after expiration leads to an immediate reduction in arterial partial pressure of O_2_ (PaO_2_) and increase in PaCO_2_, resulting in an instantaneous increase in BOLD signal. However, in breath-holding after inspiration, PaO_2_, PaCO_2_, arterial pH, and, consequently BOLD signal exhibit a biphasic change. BOLD signal initially decreases during deep inspiration, and then increases due to prolonged breath-holding [43–46].

Several studies assess CVR qualitatively, and the estimation of BOLD signal changes after vasoactive challenges can be just used for interhemispheric comparison within a subject group. To allow reliable comparison between different groups, such as healthy subjects and patients, or between different studies, data should be analyzed quantitatively. Hence, it is interesting the physiological parameter monitoring during MRI exams and further use of PaCO_2_ or PetCO_2_ to normalize BOLD responses in the following way:

(1) $$\begin{array}{c} {\text{CVR}}_{\text{BOLD}}=100\times \frac{{\text{PSC}}_{\text{hyper}}-{\text{PSC}}_{\text{normo}}}{{\text{PetC}{\text{O}}_{2}}_{\text{hyper}}-{\text{PetC}{\text{O}}_{2}}_{\text{normo}}}, \end{array}$$

where PSC_normo_ and PSC_hyper_ are the percent signal change, and PetCO_2normo_ and PetCO_2hyper_ are the end-tidal CO_2_ concentrations during measurements for normocapnia and hypercapnia, respectively.

Kastrup et al. have reported that 5% of CO_2_ caused an increase of 13 ± 2 mmHg in PetCO_2_ and 2.8 ± 0.5% in BOLD response, resulting in an CVR value of 0.21 ± 0.06%/mmHg [42]. More recently, Yezhuvath et al. have reported an CVR value of 0.31 ± 0.08%/mmHg in a study with 5% CO_2_ inhalation and PetCO_2_ measurement [70]. Preliminary data from our group showed a similar CVR value (0.29 ± 0.07%/mmHg) for twenty healthy subjects, that were also submitted to a CO_2_ inhalation protocol.

However, the reliability of CVR quantification with BOLD-MRI in response to hypercapnia can be a concern. Hence, recent studies have investigated the intersubject and interhemispheric variability, and the short-term reproducibility in groups of healthy subjects. They concluded that CVR quantification have a good between-session and interhemispheric reproducibility, making the technique useful and safe for CVR assessment in patients with cerebrovascular diseases [62, 63].

Another concern about using this technique to assess CVR is that BOLD signal depends on CBF, and also on CBV, CMRO_2_, PaO_2_, and hematocrit in a complex and not fully understood way. Recent investigations to address this issue showed that BOLD signal response to changes in PetCO_2_ is directly related to CBF measured with SPECT and ASL, in healthy subjects and in patients with steno-occlusive disease [7, 71]. Results also showed that BOLD method can differentiate impaired hemispheric CVR from normal CVR. 

Furthermore, in patients with unilateral internal carotid artery (ICA) stenosis or occlusion, CVR estimated by BOLD-MRI in the MCA territory was found to be well correlated to changes in MCA CBF velocity measured with TCD after inhalation of 3–7% CO_2_. In areas of infarction, reduced BOLD CO_2_ reactivity was observed. In noninfarcted regions ipsilateral to the stenosis or occlusion, CVR was impaired [11, 40]. In such areas, TCD was not able to estimate CVR, showing an advantage of BOLD-MRI technique. 

To prevent ischemic stroke, patients with severe carotid stenosis often benefit from interventions, such as carotid endarterectomy (CEA) and carotid angioplasty with stent placement (CAS) [72–74]. Recently, studies have reported that patients with carotid stenosis, who showed impaired CVR before carotid intervention, had great CVR improvement after CEA or CAS [5, 6]. This finding is probably due to an improvement in vasodilatory ability, indicating return of vascular reserve capacity. Although such patients may benefit from greater hemodynamic improvements, hyperperfusion is still a concern that can lead to hemorrhage [75]. In fact, a condition called cerebral hyperperfusion syndrome (CHS) after carotid intervention has been classically described as an acute neurologic deficit occurring from hours to days following a carotid procedure and represents a spectrum of clinical symptoms ranging from severe unilateral headache, to seizures and focal neurologic defects, to intracerebral hemorrhage in its most severe form. The mechanism appears to be related to increased regional cerebral blood flow secondary to loss of cerebrovascular autoregulation [76, 77]. 

It is worth noting that the above observations about the influence of CO_2_ in BOLD response and the impaired CVR in cerebrovascular diseases have important impact on fMRI brain activation studies. Since perfusion is not homogeneous throughout human brain, the statistical maps obtained in typical fMRI studies may reflect, partially, perfusional differences. Therefore, it has been recently proposed the use of hypercapnia, mainly BHT, as a way to normalize fMRI results, which would take into account individual perfusion responses [78–80]. However, the discussion of these calibration methods is not in the scope of this review.

## 4. CVR Mapping with ASL

Quantitative maps of CBF can be obtained with ASL, a noninvasive MRI technique which uses water protons presented in arterial blood as an endogenous perfusion tracer. This technique is based on the differentiation between stationary spins and spins flowing with blood, which is labeled using radiofrequency pulses. As labeled intravascular spins reach the capillaries and do exchanges with brain tissue through blood-brain barrier, tissue magnetization is altered, making it possible to obtain images in which contrast is proportional to cerebral perfusion [81–83]. There are different ASL methods, but all of them are based on the same principle. Images are acquired distally to the labeling plane after labeled blood flowed to the target tissue. Perfusion map is then obtained by subtracting labeled and control images. The latter are acquired without spin labeling.

Therefore, perfusion images with high spatial resolution showing quantitative CBF values in mL/g/min can be calculated [83], allowing detection of global and regional altered perfusion and data comparison in longitudinal studies [84]. Despite its advantages, ASL has limited signal-to-noise ratio and complex flow kinetics, requiring measurements of T1 values for blood and tissue, labeling efficiency and arterial transit time for absolute CBF quantification [85]. However, its noninvasiveness is very suitable for perfusion studies in patients with renal insufficiency, for repetitive follow-up studies and for pediatric investigations in which exogenous contrasts or radioactive tracers are not recommended. 

Furthermore, ASL can be used to assess CVR, by mapping CBF under normo- and hyper-capnic conditions [36]. CVR can be calculated as %/mmHg in the following way:

(2) $$\begin{array}{c} {\text{CVR}}_{\text{ASL}}=\frac{100}{{\text{CBF}}_{\text{normo}}}\times \frac{{\text{CBF}}_{\text{hyper}}-{\text{CBF}}_{\text{normo}}}{{{\text{PetCO}}_{2}}_{\text{hyper}}-{{\text{PetCO}}_{2}}_{\text{normo}}}, \end{array}$$

where CBF_normo_ and CBF_hyper_ are the cerebral blood flow, and PetCO_2normo_ and PetCO_2hyper_ are the end-tidal CO_2_ concentrations during measurements for normocapnia and hypercapnia, respectively.

In healthy subjects, global CBF increase was observed in response to hypercapnia [36]. Yen et al. have shown satisfactory CVR measurement reproducibility between session days and good sensitivity to small changes, which is important when investigating disease or treatment [86]. Noth et al. have compared perfusion MRI measurements at 1.5 T and 3.0 T, and observed no CBF and CVR differences between magnetic fields. CVR values in healthy subjects were 4.3 ± 0.7%/mmHg and 4.5 ± 1.3%/mmHg for 1.5 T and 3.0 T, respectively [36]. Other two recent studies showed regional difference in CVR values in healthy subjects. CVR in areas irrigated by ICA was lower than in areas irrigated by the basilar artery [87, 88].

ASL perfusion imaging has been clinically applied in the evaluation of cerebrovascular diseases [89]. Recent studies have demonstrated altered hemodynamics and regional CBF in patients with arterial stenosis [90]. As cited in previous session, Mandell et al. have found significant correlation between CVR values measured with BOLD and ASL in patients. For example, in a 57-year-old male patient with left ICA occlusion, CVR values in gray matter of the ipsilateral hemisphere were (CVR_BOLD_ = 0.22%/mmHg; CVR_ASL_ = 1.79%/mmHg) significantly lower than the values of the contralateral hemisphere (CVR_BOLD_ = 0.33%/mmHg; CVR_ASL_ = 3.35%/mmHg) [71].

More recently, Bokkers et al. performed two studies to assess CVR with ASL and ACZ administration. One study was in patients with carotid artery stenosis, and results showed decreased CVR in brain tissue supplied by the symptomatic ICA, when compared to regions supplied by the contralateral ICA and to CVR results in control subjects [88]. The other study was performed in patients that presented ICA occlusion. Again, results showed impaired CVR in brain tissue on the side of the occluded ICA [35]. Moreover, regional CBF and CVR values measured with ASL were significantly correlated with those measured with SPECT [91]. Therefore, ASL in combination with vascular challenge is able to evaluate CVR in patients with ICA stenosis or occlusion. 

## 5. Conclusions

Patients with arterial stenosis or occlusion have a high risk for ischemic stroke, which is significantly influenced by the degree of CVR impairment and the collateral flow development. Therefore, several studies have been investigating CVR to identify patients with increased risk and to better plan the treatment. For these purposes, MRI technique may play an important role as a noninvasive tool capable to assess cerebral reserve capacity in combination with a vascular challenge. 

Assessment of CVR with BOLD-MRI is an alternative to PET, SPECT, and TCD and can be easily included in clinical examinations. It has the advantage of mapping whole brain with good spatial resolution, allowing investigation of CVR regional distribution. And despite providing semiquantitative data, studies showed that the method is robust, reliable, and reproducible, allowing assessment of CVR in patients with cerebrovascular diseases. On the other hand, ASL is a quantitative method to assess whole-brain CBF and CVR. Moreover, it has been used to selectively label blood flowing through specific cerebral arteries, which allows investigation of collateral flow. Recently, CBF-ASL has been used in many clinical applications, such as in arterial steno-occlusive disease, but also in hypertension [92], diabetes [93], Alzheimer's disease [94], epilepsy [95], and brain tumor [96], in which resting perfusion is believed to be altered. However, in some cases, regional CBF is normal, but cerebral reserve capacity may be exhausted. In such cases, CVR mapping may be a powerful tool to investigate compromised hemodynamics. 

In addition to the known disadvantages of each method, other factors may interfere with the results of arterial stenosis or occlusion investigation, such as the small number of subjects in some studies [5–7, 35, 40, 90]; inclusion of both symptomatic and asymptomatic patients; inclusion of both arterial stenosis and occlusion in the same study; no investigation of other factors that alter CVR, such as age [97], gender [98], hypertension [92], and diabetes [93].

In conclusion, functional MRI techniques (BOLD and ASL) are an alternative to PET, SPECT, and TCD for the assessment of CVR and can be easily included during an MR imaging scan for clinical purposes. Those methods are robust, reliable and reproducible, allowing assessment of CVR in patients with cerebrovascular diseases. Therefore, the clinical impact of a combination of those MRI techniques as a tool to assess CVR in patients with carotid steno-occlusive disease and to select the best recanalization strategy for patients with carotid stenosis requires further clinical investigation.
